# Learning Inverse Statics Models Efficiently With Symmetry-Based Exploration

**DOI:** 10.3389/fnbot.2018.00068

**Published:** 2018-10-23

**Authors:** Rania Rayyes, Daniel Kubus, Jochen Steil

**Affiliations:** Institut für Robotik und Prozessinformatik, Technische Universität Braunschweig, Braunschweig, Germany

**Keywords:** symmetries, inverse statics models, inverse dynamics models, efficient learning, direction sampling, goal babbling

## Abstract

Learning (inverse) kinematics and dynamics models of dexterous robots for the entire action or observation space is challenging and costly. Sampling the entire space is usually intractable in terms of time, tear, and wear. We propose an efficient approach to learn inverse statics models—primarily for gravity compensation—by exploring only a small part of the configuration space and exploiting the symmetry properties of the inverse statics mapping. In particular, there exist symmetric configurations that require the same absolute motor torques to be maintained. We show that those symmetric configurations can be discovered, the functional relations between them can be successfully learned and exploited to generate multiple training samples from one sampled configuration-torque pair. This strategy drastically reduces the number of samples required for learning inverse statics models. Moreover, we demonstrate that exploiting symmetries for learning inverse statics models is a generally applicable strategy for online and offline learning algorithms. We exemplify this by two different learning approaches. First, we modify the Direction Sampling approach for learning inverse statics models online, in a plain exploratory fashion, from scratch and without using a closed-loop controller. Second, we show that inverse statics mappings can be efficiently learned offline utilizing lattice sampling. Results for a 2R planar robot and a 3R simplified human arm demonstrate that their inverse statics mappings can be learned successfully for the entire configuration space. Furthermore, we demonstrate that the number of samples required for learning inverse statics mappings for 2R and 3R manipulators can be reduced at least by factors of approximately 8 and 16, respectively–depending on the number of discovered symmetries.

## 1. Introduction

The learning of motor capacities and skills has always been a core topic of the developmental approach to robot cognition (Asada et al., 2001), as mastering the body is fundamental for any embodied agent. Since the seminal work on human motor control in the 1990th (Wolpert and Kawato, 1998; Wolpert et al., 1998), it is widely believed that forward and inverse models play a crucial role in the motor control architectures. Numerous learning schemes have been proposed during the last decades for exploratory learning of robot forward and inverse kinematics, where in the developmental context exploratory learning without the initial constraint of a particular task or trajectory is the main focus. Note that in the latter case more specialized schemes can be applied both for kinematics (D'Souza et al., 2001) and dynamics (Peters and Schaal, 2008; Meier et al., 2016) and the learning problem is locally convex which simplifies the task significantly. Motor control for the entire configuration space, however, remains a major challenge because sampling the entire action or observation space is usually very costly and the non-convexity of the model (e.g., due to kinematic redundancy) poses additional problems.

Efficiency is one of the major challenges in learning (inverse) kinematics and dynamics models. Reducing the number of required samples to learn these models in practical experiments is beneficial regarding time and hardware costs. We therefore propose *symmetry-based exploration* to effectively reduce the number of required samples. This can be done by exploiting the mapping properties to learn a model that is valid for the entire action/observation space. For example, it is a particular property of inverse statics maps (ISMs) (i.e., the map that assigns a required static torque to maintain a desired joint configuration of the robot) that multiple configurations require the same absolute static torque to be maintained. We denote this configuration set as symmetry set. We exploit the functional relation between configurations in the symmetry set to show that learning ISMs can be done very efficiently by exploring only one configuration and learning the corresponding symmetric configurations. To this aim, we propose a scheme to discover and learn symmetries, and then we exploit these symmetries to drastically reduce the number of required samples regardless of the particular learning scheme. The paper demonstrates the generic nature of the symmetry concept to accelerate the learning process through exploiting symmetries with different learning schemes online and offline.

Learning ISMs has previously been done offline only and by using a feedback-controller to collect samples and to enhance an already existing model (e.g., Luca and Panzieri, 1993; Xie et al., 2008). In this paper, Direction Sampling (Rolf, 2013), which has been previously proposed as an extension of Goal Babbling (Rolf et al., 2011) to learn inverse kinematics (IK), is modified to learn ISMs also online, from scratch and without using any controller in a plain exploratory fashion. Learning ISMs in an exploratory fashion is challenging as the straightforward application of random torques bears the risk to destroy any manipulator if no further safety layers are present and to respect joint-wise torque limits alone does not solve this problem, other than in kinematics, where joints limits can be enforced easily and without endangering the robot hardware. Hence, the exploration may yield inadmissible torques which result in accelerating the robot manipulator and the robot hitting its joint limits^1^. Consequently, the learner will be disturbed because of the resulting invalid training sample consisting of inadmissible torque which is not corresponding to the joint limits' configuration where the robot settles in. To avoid this situation, torque combination limits must be considered in addition to the joint-wise torque limits. We therefore explore and learn the set of admissible static torques to overcome this issue as explained in detail in section 5.1.

These aforementioned challenges also illustrate more restrictions and difficulties of learning ISMs in comparison to learning IK. For example, the application of a torque produces dynamics, other than in the kinematics domain where application of a joint command can be treated as instantaneously effective, because the underlying joint controllers hide and control the dynamics. Furthermore, the training samples in IK are always valid samples since the end-effector pose always corresponds to a valid robot configuration even when the robot hits its joint limits, which is not the case in ISMs. Moreover, IK usually maps from Cartesian (observation) space to configuration (action) space, i.e., from a lower dimensional space to a higher dimensional one, while the dimensions of observation and action spaces in ISMs are usually identical since ISMs map from configuration (observation) space to motor (action) space. Learning the mapping between spaces with identical dimensions is more difficult as both dimensions scale with the number of DoFs. Consequently, more samples are required to learn the model in contrast to IK. Hence, exploiting symmetries and exploring only a small part of the configuration space is also motivated to mitigate the curse of dimensionality problem. It reduces the number of required samples as the efficiency factor increases for higher DoFs. For instance, it increases to 8 for a 2R planar manipulator and to 16 for a 3R robot manipulator as illustrated in section 7.

The remainder of the paper is structured as follows: Section 2 reviews related work. Section 3 introduces the concept of symmetries. Section 4 explains symmetry discovery and symmetry exploitation in learning. Section 5 addresses learning ISMs online and explains the proposed Constrained Direction Sampling. Lattice sampling is introduced briefly in section 6. Section 7 presents experimental results and the efficiency gained by exploiting symmetries for learning ISMs which is illustrated by Constrained Direction Sampling (online) and a batch learning technique using lattice sampling for a 2R and a 3R manipulators. Section 8 concludes the work.

## 2. Related work

Our main goal is increasing the efficiency of learning models, in particular for learning inverse statics. As learning ISMs has been done previously only offline, we modified the Direction Sampling method (Rolf, 2013) for learning ISMs online as well. This paper therefore discusses three major points: learning efficiently, learning inverse statics models, and online goal-directed approaches. This section presents the previous related work.

### 2.1. Learning efficiently

Various approaches have previously been proposed for tackling the efficiency problem of learning. Some previous research proposed exploring the observation space instead of the action space to avoid the curse of dimensionality. For instance, learning IK by exploring the observation space (Cartesian space) and learning only one configuration for each pose to mimic infants efficient sensorimotor learning (e.g., Rolf et al., 2011; Rolf and Steil, 2014; Rayyes and Steil, 2016) instead of learning forward kinematics mappings by exploring the higher dimensional action space (configuration space) e.g., Motor Babbling (Demiris and Meltzoff, 2008).

Other research proposed that online learning of inverse models can be done in part of the workspace only in order to increase the efficiency and reduce the number of required samples (Rolf et al., 2011; Baranes and Oudeyer, 2013), since online learning approaches have the tendency to require more samples than offline methods. Efficient exploration by efficient sampling (active policy iteration) was proposed in Akiyama et al. (2010), however it has been proposed for batch learning only. Efficient learning has been also addressed for solving different tasks (e.g., Şimşek and Barto, 2006) based on Markov Decision Process and reward function. In this paper, we propose *symmetry-based exploration* to learn ISMs for the entire configuration space effectively by exploring a small part of it and exploiting the symmetries of ISMs which reduces the number of required samples. The proposed strategy is applicable for online and offline learning schemes.

### 2.2. Learning inverse statics models

Compensating forces and torques due to gravity is very important for advanced model-based robot control. The gravitational terms of the inverse dynamics models are usually computed either by estimating inertial parameters of the links or from CAD data of the robot. However, if no appropriate model exists e.g., for advanced complex robots or for soft robots, or if no prior knowledge on the inertial parameters of the links is available, *learning* these gravitational terms is a promising option. Previous research on learning ISMs has been done offline using a closed-loop controller to collect training data and often to enhanced existing (parametric) models (e.g., Luca and Panzieri, 1993; Xie et al., 2008). Early data-driven gravity compensation approaches are based on iterative procedures for end-point regulation (De Luca and Panzieri, 1994; De Luca and Panzieri, 1996). Recent works (Giorelli et al., 2015; Thuruthel et al., 2016b) have proposed data-driven learning techniques to control the end-point of continuum robots in task space. Where ISMs map between the desired end effector poses and the cable tensions. However, feedback controllers and inefficient Motor Babbling were implemented to obtain the training data and to learn ISMs offline only. In contrast, we propose learning ISMs online, in an exploratory fashion, from scratch and without using a closed-loop controller. Besides, we exploit the symmetry properties of ISMs to learn ISMs efficiently online and offline for the entire configuration space.

### 2.3. Goal babbling and direction sampling

Various schemes have been proposed to replicate human movement skill learning and human motor control based on internal models (Wolpert et al., 1998), i.e., learning forward models (e.g., Motor Babbling Demiris and Meltzoff, 2008), and inverse models (e.g., distal teachers Jordan and Rumelhart, 1992 and feedback error learning; Gomi and Kawato, 1993). In contrast to Motor Babbling where the robot executes random motor commands and the outcomes are observed, there is evidence that even infants do not behave randomly but rather demonstrate goal-directed motion already few days after birth (von Hofsten, 1982). They learn how to reach by trying to reach and they iterate their trails to adapt their motion. Hence, Goal Babbling was proposed and inspired by infant motor learning skills for direct learning of IK within a few 100 samples (Rolf et al., 2010, 2011). Various other schemes were proposed for learning IK e.g., direct learning of IK (D'Souza et al., 2001; Thuruthel et al., 2016a) and incremental learning of IK (Vijayakumar et al., 2005; Baranes and Oudeyer, 2013).

To apply Goal Babbling, a set of predefined targets, e.g., a set of positions to be reached, is required and then used to obtain the IK which is valid only in the predefined area. Direction Sampling (Rolf, 2013) has been proposed as an extension of Goal Babbling, to overcome the need for predefined targets and gradually discover the entire workspace. The targets are generated while exploring and the IK is learned simultaneously. In previous work, we already illustrated the scalability of online Goal Babbling with Direction Sampling in higher dimensional sensorimotor spaces up to 9-DoF COMAN floating-base (Rayyes and Steil, 2016). Goal Babbling has also been extended to learn IK in restricted areas (Loviken and Hemion, 2017) and to other domains e.g., speech production (Moulin-Frier et al., 2013; Philippsen et al., 2016) and tool usage (Forestier and Oudeyer, 2016). Besides, it has been also applied to soft robots (Rolf and Steil, 2014). However, it is striking that none of these schemes have been extended or transferred to learn the forward or inverse dynamics. As Goal Babbling shows high scalability and adaptability in "learning while behaving" fashion, we focus in this paper on learning ISMs, as a first step in the direction of exploratory dynamics leaning, by modifying the previously proposed Direction Sampling based on online Goal Babbling.

## 3. Inverse static models and symmetric configurations

In this section, we first explore fundamental properties of ISMs, subsequently devise the concept of symmetries and then define the notion of primary and secondary symmetric configurations which are finally illustrated with a 2R planar manipulator. We will use the term torques instead of generalized actuator forces as our main target are manipulators with revolute joints only.

### 3.1. Properties of inverse statics maps

**Figure 1:**
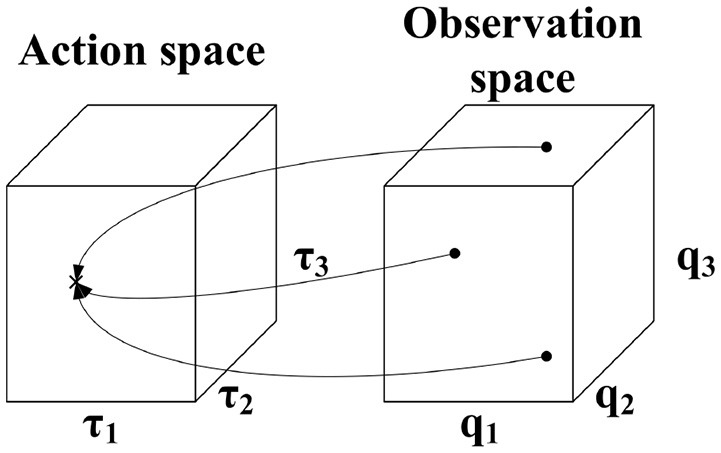
Characteristics of ISMs. The same torque is required to maintain different configurations.

ISMs map from configuration space, which constitutes the observation space, to motor space, which represents the action space. The dimensionality of the domain and codomain in ISMs are therefore identical. ISMs are many-to-one mappings, i.e., multiple configurations require the same torque to be maintained as illustrated in Figure 1.

We aim to learn the map **G** which assigns to each joint configuration $\text{q}\in Q_{p}$ a torque $\text{τ}\in T_{s}$ required to maintain this configuration:

(1) $$\begin{array}{r} \text{G}:Q_{p}\to T_{s},\;\text{G}\left(\text{q}\right)=\text{τ} \end{array}$$

$Q_{p}$ is the set of permissible configurations while $T_{s}$ is the set of required static torques to maintain these configurations. **G** typically associates each member of the set $T_{s}$ with more than one member of the domain $Q_{p}$. There typically exist respective level sets

(2) $$\begin{array}{r} L_{\text{τ}}=\left\{\text{q}:\text{G}\left(\text{q}\right)=\text{τ}\right\} \end{array}$$

with cardinalities $|L_{\text{τ}}|>1$ for admissible torque vectors $\text{τ}\in T_{s}$.

### 3.2. Symmetric configurations

We define the concept of symmetries as following:

Consider two level sets $L_{{\text{τ}}_{i}}$ and $L_{{\text{τ}}_{j}}$ where

(3) $$\begin{array}{r} {\text{τ}}_{i}=\text{Υ}{\text{τ}}_{j},\text{Υ}=\text{diag }\left(\delta_{1},\ldots ,\delta_{n}\right), \end{array}$$

$$\begin{array}{r} \delta_{k}=\pm 1,\;\overset{n}{\underset{k=1}{\sum }}\delta_{k}<n \end{array}$$

i.e., the elements in ***τ***_*i*_ and ***τ***_*j*_ differ w.r.t. their sign. Here, *n* denotes the number of DoFs and diag (δ_1_, …, δ_*n*_) denotes a diagonal matrix with δ_1_, …, δ_*n*_ on its main diagonal. We define ${\overset{}{L}}_{\text{τ}}$ as

(4) $${\overset{˘}{ℒ}}_{\tau }=\overset{2^{n}}{\underset{k\;=\;1}{\cup }}{ℒ}_{\tau_{k}}$$

${\overset{}{L}}_{\text{τ}}$ is the union of all level sets fulfilling Equation (3), i.e., the union of the level sets which have the same absolute value of the elements in the torque vector.

Two classes of configurations in these level sets can be distinguished. Primary symmetric configurations, also denoted as primary symmetries, constitute those pairs of configurations ${\text{q}}_{r},{\text{q}}_{s}\in {\overset{}{L}}_{\text{τ}}$ for which

(5) $$\begin{array}{r} {\text{M}}_{r,s}{\text{q}}_{r}+{\text{N}}_{r,s}{\text{q}}_{s}={\text{d}}_{r,s} \end{array}$$

holds – where ${\text{d}}_{r,s}\in {\mathbb{R}}^{n}$ and ${\text{M}}_{r,s},{\text{N}}_{r,s}\in {\mathbb{R}}^{n\times n}$ are constant (in particular independent of the choice of ***τ***). The set of all configurations in ${\overset{}{L}}_{\text{τ}}$ which are directly or transitively related by Equation (5) is called the set of primary symmetries (SPS) denoted by $S\subset {\overset{}{L}}_{\text{τ}}$.

Secondary symmetric configurations, also denoted as secondary symmetries, constitute those configurations in ${\overset{}{L}}_{\text{τ}}$ for which at least one of ***d***_*r, s*_, ***M***_*r, s*_, ***N***_*r, s*_ is a function of ***q*** and/or ***τ***.

#### 3.2.1. Symmetric configurations of a planar 2R manipulator

**Figure 2:**
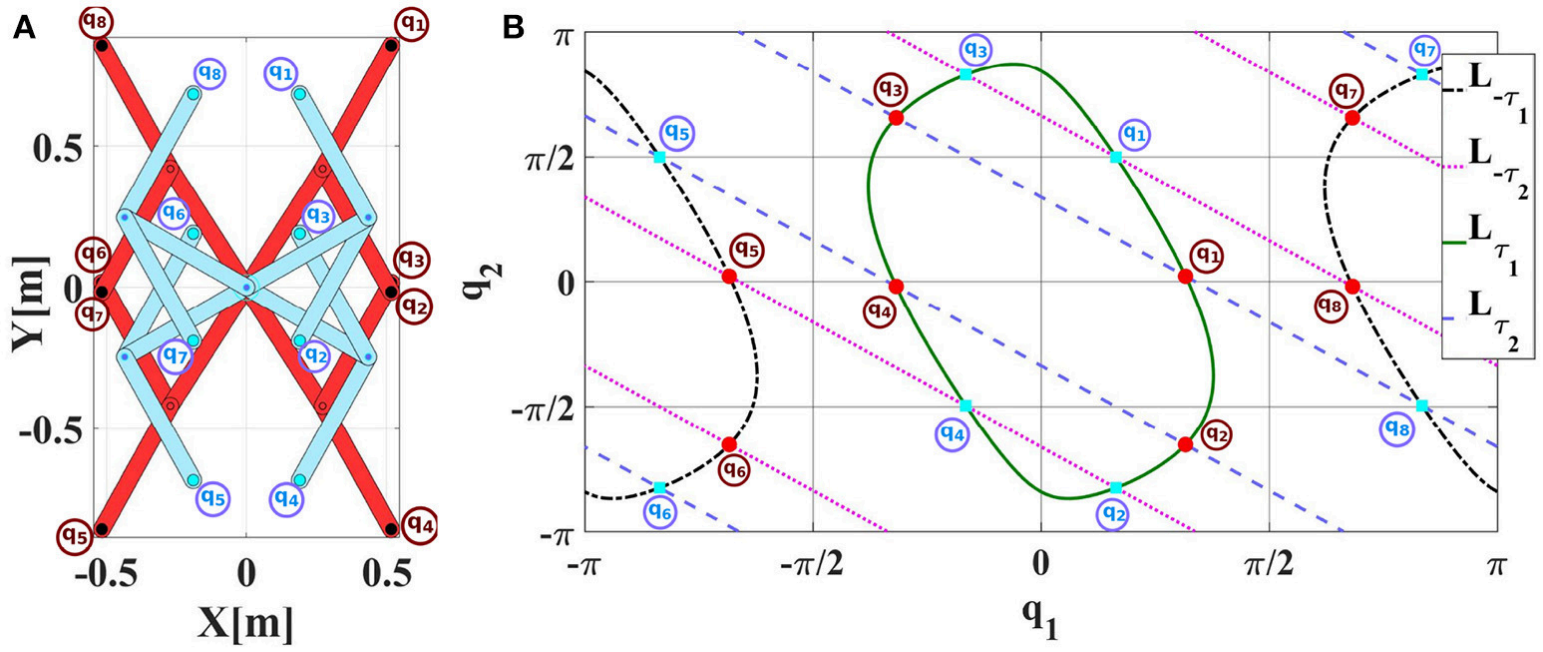
**(A)** Symmetric configurations of a 2R planar robot which require the same absolute static torque to be maintained. Configuration pairs in each configuration set illustrated in blue $S_{A}$ (and red $S_{B}$, respectively) are primary symmetric to each other in the same set. The two sets are secondary symmetric to each other. Note that the manipulator is stretched out to the right in its zero configuration and that the gravity vector points downwards into negative y-direction. **(B)** Component-wise level sets $L_{\tau_{1}}$,$L_{\tau_{2}}$,$L_{-\tau_{1}}$,$L_{-\tau_{2}}$ of the 2R planar manipulator. The 16 intersection points constitute symmetric configurations. Their colors and numbers correspond to the configurations shown in Figure 2A. The numbers are based on Equation (11).

To exemplify the idea of primary symmetries and secondary symmetries, Figure 2A shows all symmetric configurations of a 2R planar robot. There are 16 configurations which need the same absolute static torque to be maintained and they can be separated into two disjoint sets $S_{A}$ (blue) and $S_{B}$ (red) of 8 configurations each.

The set $S_{A}$ constitutes a set of primary symmetries. The symmetric configurations in $S_{A}$ are also geometrically symmetric as illustrated in Figure 2A, it is therefore, easy to find the functional relation between them with the linear equation given in Equation (5). Similarly, the set $S_{B}$ constitutes a set of primary symmetries as well. These two sets are secondary symmetric to each other as $S_{A}$ and $S_{B}$ have identical absolute static torques. The secondary symmetries occur by relating configurations from $S_{A}$ with those from $S_{B}$, however; there is no simple closed form functional relations between these two sets. We will therefore consider only primary symmetries in our experimental results.

For visualization purposes, we use component-wise level sets for the 2R planar manipulator (cf. Figure 2A) as defined below and illustrated in Figure 2B:

(6) $$\begin{array}{l} {ℒ}_{\tau_{1}}=\left\{q:G(q)={\left[\tau_{1},\tau_{2}\right]}^{T},\tau_{2}\in \mathbb{R}\right\},\\ {ℒ}_{-\tau_{1}}=\left\{q:G(q)={\left[-\tau_{1},\tau_{2}\right]}^{T},\tau_{2}\in \mathbb{R}\right\} \end{array}$$

(7) $$\begin{array}{l} {ℒ}_{\tau_{2}}=\left\{q:G(q)={\left[\tau_{1},\tau_{2}\right]}^{T},\tau_{1}\in \mathbb{R}\right\},\\ {ℒ}_{-\tau_{2}}=\left\{q:G(q)={\left[\tau_{1},-\tau_{2}\right]}^{T},\tau_{1}\in \mathbb{R}\right\} \end{array}$$

$L_{\pm \tau_{1}}$ and $L_{\pm \tau_{2}}$ fix one component of ***τ*** while the other one is not restricted. All pairwise intersection points of component-wise level sets $L_{\pm \tau_{1}}$ and $L_{\pm \tau_{2}}$ constitute symmetric configurations as they have the same absolute values of the elements in the torque vectors and hence fulfill Equations (2, 3).

Note that the component-wise level set is different from the level set which is defined in Equation (2). The component-wise level set fixes only one component of ***τ***, while the level set in Equation (2) fixes all components of ***τ***. Based on Equations (2–4), the level sets for the 2R robot illustrated in Figure 2A are:

(8) $$\begin{array}{l} \;{\overset{˘}{ℒ}}_{\tau }=\overset{2^{2}}{\underset{k\;=\;1}{\cup }}{ℒ}_{\tau_{k}}\\ {ℒ}_{\tau_{1}}=\left\{q:G(q)={\left[+\tau_{1},+\tau_{2}\right]}^{T}\right\}\\ {ℒ}_{\tau_{2}}=\left\{q:G(q)={\left[+\tau_{1},-\tau_{2}\right]}^{T}\right\}\\ {ℒ}_{\tau_{3}}=\left\{q:G(q)={\left[-\tau_{1},+\tau_{2}\right]}^{T}\right\}\\ {ℒ}_{\tau_{4}}=\left\{q:G(q)={\left[-\tau_{1},-\tau_{2}\right]}^{T}\right\} \end{array}\}$$

Each level set comprises 4 configurations corresponding to 4 points in the pairwise intersections of the component-wise level sets in Figure 2B. Therefore, the symmetric configurations form the union of the level sets ${\overset{}{L}}_{\text{τ}}$ and the pairwise intersections of component-wise level sets ${⋂}_{i=1}^{2^{2}}L_{\tau_{i}}$.

Like the configurations in Figure 2A, the 16 intersection points in Figure 2B can be separated into the two disjoint sets $S_{A}$ and $S_{B}$ indicated by the color of the points. The numbers indicate the corresponding torque (intersection point) for each configuration in Figure 2A which fulfill Equation (11) as well. We can also derive the required torque for each joint geometrically from Figure 2A and relate it with Figure 2B. Following the right-hand rule, we can detect the sign of the torque for each joint. In this setup, the zero configuration is where the arm stretched out to the right. Every torque of a joint whose link is located on the right side of a virtual vertical line/plane will have a positive sign. For instance, for ***q***_**1**_ in $S_{B}$ (red), we can imagine a vertical line passing through the origin and a second vertical line passing through the second joint axis. Both links are on the right side of the lines so their torques are positive. On the contrary, both links of ***q***_**8**_ in $S_{B}$ (red) are on the left side of the imaginary vertical lines. So their torques are negative.

## 4. Accelerating learning by exploiting symmetries

Each torque vector ***τ*** with identical absolute values of its elements corresponds to a non-singleton set ${\overset{}{L}}_{\text{τ}}$ of configurations. Hence, functional relations between the configurations in ${\overset{}{L}}_{\text{τ}}$ can be exploited to generate training data and associate each configuration in ${\overset{}{L}}_{\text{τ}}$ with its applied torque vector ***Υ***′***τ*** by observing just one configuration from ${\overset{}{L}}_{\text{τ}}$ where

(9) $$\begin{array}{r} {\text{Υ}}^{\prime }=\text{diag }\left(\delta_{1},\ldots ,\delta_{n}\right) \end{array}$$

$$\begin{array}{r} \delta_{k}=\pm 1,\;\overset{n}{\underset{k=1}{\sum }}\delta_{k}\leq n \end{array}$$

Before symmetric configurations can be exploited in this way, they need to be discovered and the functional relations between them need to be learned or inferred. Symmetric configurations can be discovered by applying suitable torque profiles to the manipulator (cf. section 4.1). Once a number of *n*_*sym*_ functional relations is determined, each applied motor command ***τ***_*i*_ generates a sample (***q***_*i*_, ***τ***_*i*_) as well as *n*_*sym*_−1 further samples $\left({\text{q}}_{j},{\text{Υ}}_{i}^{\prime }{\text{τ}}_{i}\right),i\neq j$ obtained by evaluating the previously established functional relations between symmetric configurations which are explained in section 4.2. Increasing the efficiency by exploiting symmetries and limiting the exploration to only one part of configuration space is explained in section 4.3.

### 4.1. Discovering symmetric configurations

For symmetry discovery, sequences of suitable torque profiles are applied with the same absolute starting and ending torque values. Algorithm 1 shows the required steps for discovering the symmetries associated with a single torque vector τ^*^.

**Algorithm 1:**
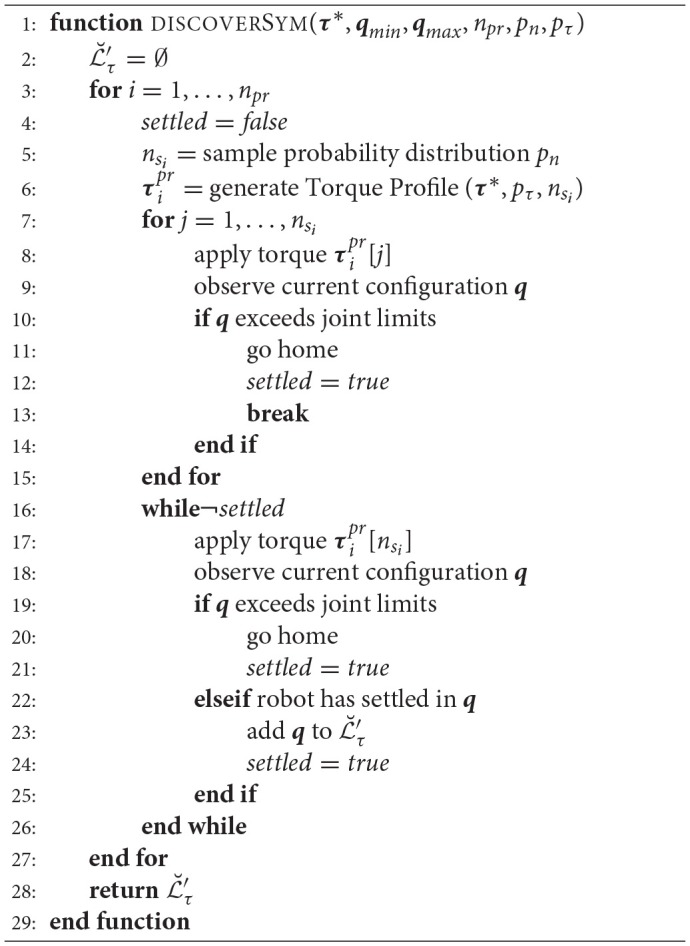
Symmetry Discovery using Torque Profiles

**Figure 3:**
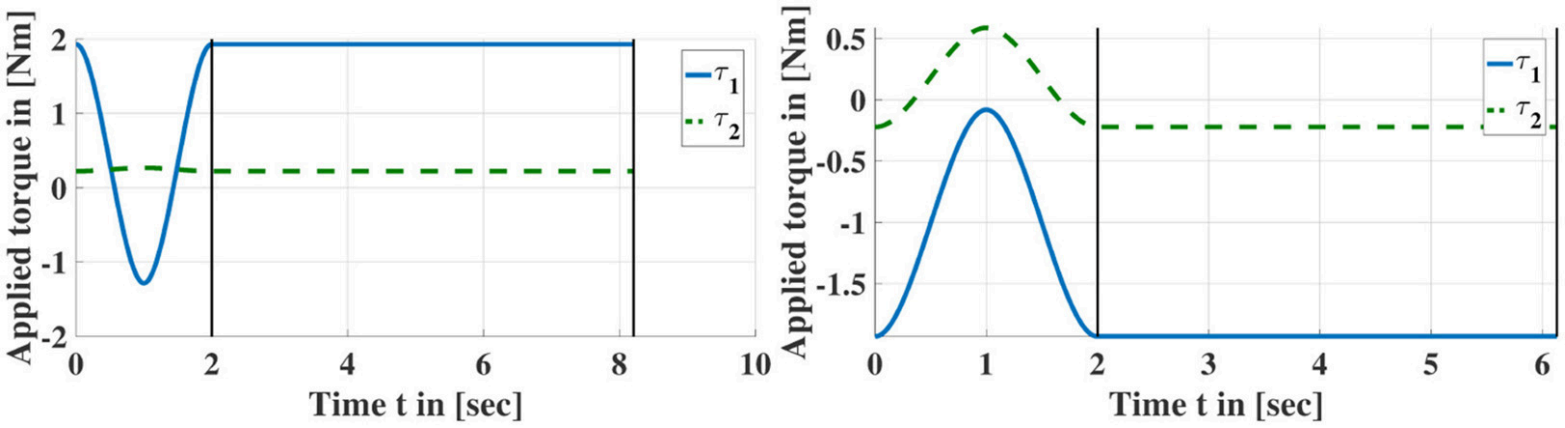
Examples of torque profiles for symmetry discovery. First, a torque spline is applied with the same initial and terminal absolute torque values. Subsequently, a constant torque is commanded until the manipulator settles at a configuration.

Let ***τ***^*pr*^ denote a torque profile. Starting from the home configuration ***q***^*home*^, a number *n*_*pr*_ of torque profiles ${\text{τ}}_{i}^{pr}$ are generated using splines (cf. Figure 3) and applied sequentially, where ${\text{τ}}_{i}^{pr}$ is the ith torque profile. Each torque profile has *k* = 1, .., *n*_*s*_*i*__ time steps. These torques profiles are applied with start and end-point constraints on their derivatives, i.e., ${\overset{{}^{\circ}}{\text{τ}}}_{i}^{pr}\left[1\right]={\overset{{}^{\circ}}{\text{τ}}}_{i}^{pr}\left[n_{s_{i}}\right]=\text{0}$, which is required to obtain a smooth trajectory.

For each torque profile ${\text{τ}}_{i}^{pr}$, ${\text{τ}}_{i}^{pr}\left[1\right]={\text{Υ}}^{\prime }{\text{τ}}^{*}\wedge {\text{τ}}_{i}^{pr}\left[n_{s_{i}}\right]={\text{Υ}}^{\prime }{\text{τ}}^{*}$ holds. Probability distributions *p*_*n*_ and *p*_τ_ are utilized to draw *n*_*s*_*i*__ samples and to generate intermediate torques in each profile, respectively.

After successful application of a torque profile, ${\text{τ}}_{i}^{p}\left[n_{s_{i}}\right]$ is applied as long as the manipulator has not settled yet, i.e., ${\text{τ}}_{i}^{p}\left[n_{s_{i}}\right]$ is applied until the manipulator stops moving. By reverting to the same torque magnitude at the end of each profile but applying different intermediate torques, a primary or secondary symmetric configuration can be reached. If the manipulator settles in a valid configuration, this configuration ***q*** is recorded and added to the discovered set ${\overset{}{L}}_{\tau }^{\prime }$ (if is not already contained in it) associated with the torque ***Υ***′τ^*^ and the sequence is continued with the next profile. If the manipulator reaches its joint limits during or after application of a torque profile, it goes back to its home configuration ***q***^*home*^ and the sequence is continued with the next profile. The discovered symmetries are marked as primary symmetries if they can be related according to Equation (5).

**Figure 4:**
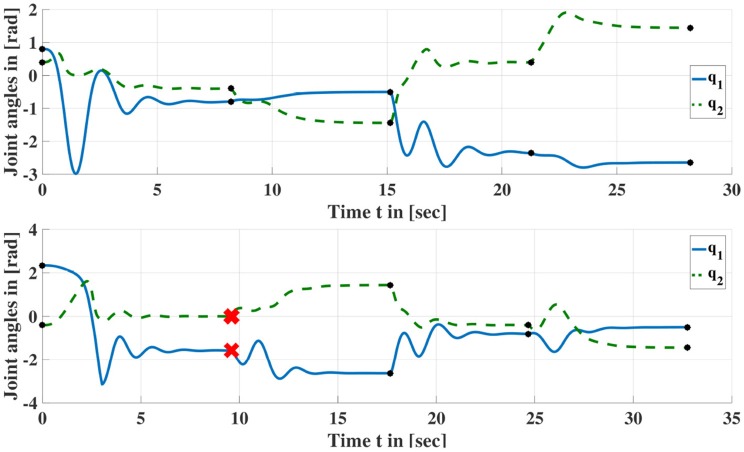
Joint trajectories resulting from applying sequences of torque profiles according to Figure 3. Red crosses indicate that the joint limits have been reached and the manipulator returned to its home configuration. Black dots indicate the end of a profile where ${\text{τ}}_{i}^{pr}\left[n_{s_{i}}\right]$ is applied until the manipulator has settled down in this configuration i.e., the manipulator has stopped moving. The corresponding configurations are entered into ${\overset{}{L}}_{\tau }^{\prime }$ (cf. Algorithm 1).

Figure 3 shows exemplary torque profiles and Figure 4 shows two joint trajectories resulting from the application of such torque profiles. 5 and 4 symmetric configurations are discovered, respectively including the initial configurations. Note that *n*_*pr*_ depends on the geometrical structure and the number of joints of the robot.

### 4.2. The functional relations between symmetric configurations

The functional relations between the primary symmetries according to Equation (5) can be determined by established multiple linear regression techniques (cf. e.g., Draper and Smith, 1998). These learned relations can then be utilized to compute the symmetric configurations for each observed ***q*** with the corresponding ***τ*** required to maintain it.

When some geometrical information about the manipulator is available and when the primary symmetries are also geometrically symmetric to each other, then the functional relations between them are easily inferred utilizing the functional relations of geometrical symmetries.

For example, the functional relations between primary symmetries for the 2R planar robot illustrated in Figure 2A are given in Equations (10, 11) according to Equation (5) and applying elementary geometric considerations:

(10) $$\begin{array}{r} S=\left\{{\text{q}}_{1},{\text{q}}_{2},{\text{q}}_{3},{\text{q}}_{4},{\text{q}}_{5},{\text{q}}_{6},{\text{q}}_{7},{\text{q}}_{8}\right\} \end{array}$$

(11) $$\begin{array}{l} \;q_{1}=[q_{1},q_{2}]\\ \;q_{2}=[q_{1},-q^{*}-q_{1}]\\ \;q_{3}=[-q_{1},q^{*}+q_{1}]\\ \;q_{4}=[-q_{1},-q^{*}+q_{1}]\\ \;q_{5}=[q_{1}+\pi ,q^{*}-q_{1}]\\ \;q_{6}=[q_{1}+\pi ,-q^{*}-q_{1}]\\ \;q_{7}=[-q_{1}-\pi ,q^{*}+q_{1}]\\ q_{8}=[-q_{1}-\pi ,-q^{*}+q_{1}]\\ \;q^{*}=q_{1}+q_{2} \end{array}\}$$

**Figure 5:**
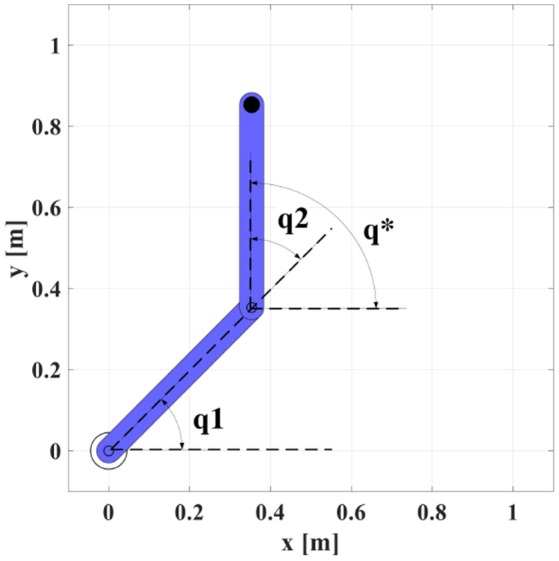
The physical and virtual joint angles of a 2R manipulator to calculate the set of primary symmetries S.

S is the set of primary symmetries, {***q***_1_, ***q***_2_, …***q***_8_} are the symmetric robot configurations, *q*_1_, *q*_2_ are the robot joint angles and q^*^ is a virtual joint angle illustrated in Figure 5.

### 4.3. Increasing efficiency by exploiting symmetries

#### 4.3.1. Bijective configuration-torque set (**BCTS**)

**Figure 6:**
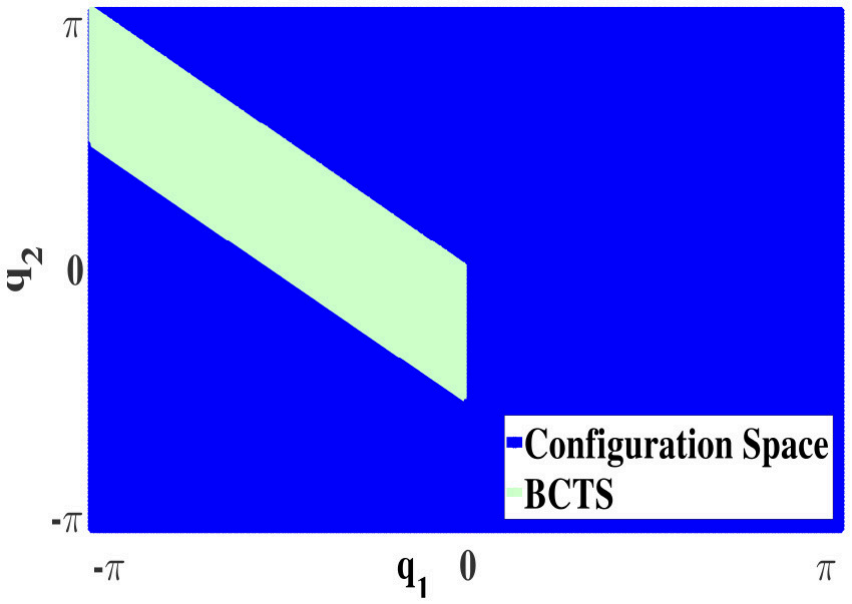
**BCTS** in the configuration space for a 2R planar manipulator.

Owing to the symmetry properties of ISMs, only a fraction of the configuration space needs to be explored. We denote this subspace as bijective configuration-torque set (**BCTS**). The **BCTS** is a set of configurations which contains exactly one unique configuration ***q*** for each admissible absolute static torque ***τ***. **BCTS** is determined based on the set of primary symmetries. For example, Figure 6 illustrates the **BCTS** (green area) for the 2R planar robot (cf. Figure 2A) which is determined based on the set of primary symmetries S given in Equations (10, 11).

As configurations outside the **BCTS** are symmetric to those inside the **BCTS**, ISMs can be learned for the entire configuration space by exploring merely the **BCTS** and exploiting the functional relations between symmetries. Constraining the exploration to discover the **BCTS** only increases the efficiency of learning and decreases the number of required samples to learn ISMs as we explore non-symmetric samples only.

For the 2R planar robot shown in Figure 2A, the currently achievable reduction factor *r* w.r.t. required samples is *r* = 8 as the primary symmetry set has cardinality 8, while exploiting secondary symmetries would further increase *r* up to 16. For the 3R simplified human arm (Babiarz et al., 2015) illustrated in Figure 9, the cardinality of the primary symmetry set increases to *r* = 16. Exploiting secondary symmetries would again yield far higher reduction factors depending on the properties of the manipulator, however, we currently have no means to exploit them.

## 5. Learning inverse static models online

In order to learn ISMs for the entire configuration space online, from scratch, in a plain exploratory fashion and without using a feedback controller , we employ Direction Sampling (Rolf, 2013). However, to apply it successfully for bootstrapping ISMs, several modifications to the original scheme are necessary. We therefore propose Constrained Direction Sampling. First, the constraint in form of the set of statically admissible torques is introduced.

### 5.1. Set of static torques (SST)

In the established Goal Babbling and Direction Sampling (Rolf et al., 2011; Rolf, 2013; Rayyes and Steil, 2016), exploratory noise is added in the action space in order to explore and learn new configurations. However, adding this exploratory noise to motor commands (torques) in ISMs may yield inadmissible torques. Consequently, the robot will accelerate and hit its joint limits which results in invalid training samples (inadmissible torques which don't correspond to the joint limits' configuration where the robot settles in).

In order to avoid such situations, the set of statically admissible torques (**SST**) should be estimated beforehand or learned and the exploration should be constrained to the **SST**. Therefore, we modify Goal Babbling and Direction Sampling in this paper to limit the exploration to this set with applying the nearest neighbor strategy. These modified approaches are termed Constrained Goal Babbling and Constrained Direction Sampling, respectively.

The set of statically admissible torques (**SST**) is defined as:

(12) $$\begin{array}{r} T_{s}=\left\{\text{τ}|\exists \text{q}\in Q_{p}:\text{τ}-\text{G}\left(\text{q}\right)=0\right\} \end{array}$$

**Figure 7:**
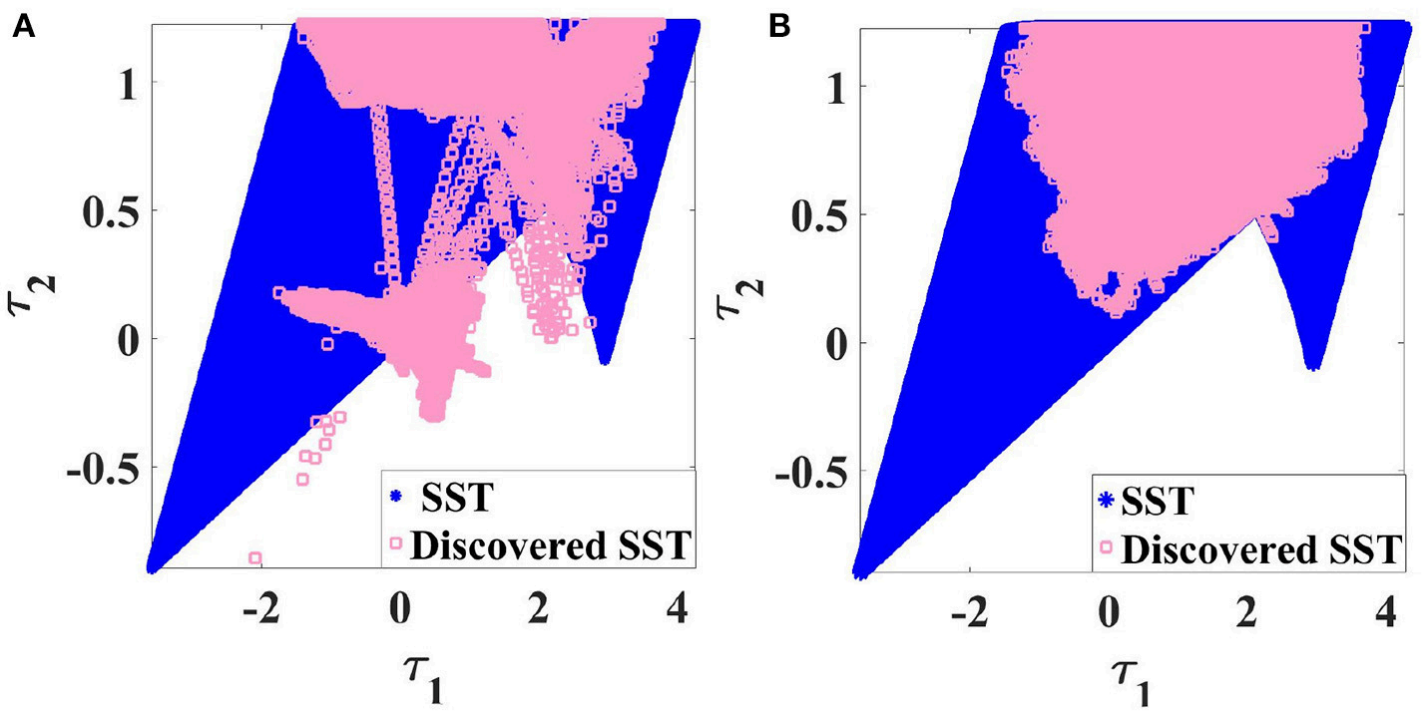
Discovered **SST** of a 2R planar manipulator with specific joint limits **(A)** with original Goal Babbling, **(B)** with Constrained Goal Babbling.

Each time the robot hits its joint limits during the learning process, the corresponding torque is marked as inadmissible and the **SST** estimate is updated accordingly. Delaunay triangulation is used to estimate the **SST** boundary. Exploratory noise (cf. Equation 14) will be added to the static torque and the nearest neighbor algorithm is employed to assign each invalid torque to a valid one before execution. Figure 7A shows the **SST** (blue points) for a 2R planar manipulator with specific joint limits and illustrates that applying the original Goal Babbling and adding explanatory noise might result in torques outside the **SST** i.e., inadmissible torques. After applying Constrained Goal Babbling, the exploration is limited to the **SST** as illustrated in Figure 7B; this avoids generating invalid training samples and avoids the robot hitting its joints limits as well. To save time, this exploration can be performed in conjunction with symmetry discovery as detailed in section 5.4.

### 5.2. Constrained direction sampling for learning ISMs

Originally, Direction Sampling was proposed in Rolf (2013) to learn IK. In this paper, we modify Direction Sampling to learn ISMs by incorporating **SST** constraints and the nearest neighbor strategy. Moreover, our approach can be applied to robots with both prismatic and revolute joints. Algorithm 2 shows the individual steps of the Constrained Direction Sampling. The initial inverse estimate ***Ĝ***(***q***) at time instant *t* = 0 yields some constant default torque ***Ĝ***(***q***) = ***τ***^*home*^ corresponding to some comfortable default configuration (home posture) ***q***^*home*^ (cf. line 2 in Algorithm 2). The robot starts exploring from its home posture ***q***^*home*^ and the targets are generated along a random direction ***Δq*** as given in Equation (13):

(13) $${\text{q}}_{t}^{*}={\text{q}}_{t-1}^{*}+\frac{\text{ε}}{\left\|{\text{w}}^{T}\text{Δ}\text{q}\right\|}\cdot \;\text{Δ}q$$

**Algorithm 2:**
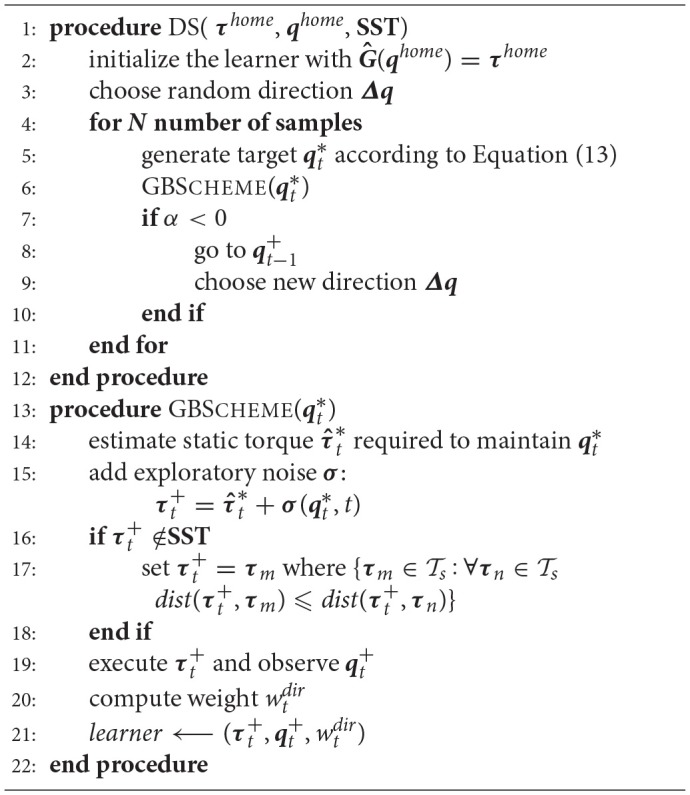
Constrained Direction Sampling

where ${\text{q}}_{t}^{*}$ is the currently generated target, ${\text{q}}_{t-1}^{*}$ is the previous one, ***w*** is a weighting vector as the joint space may be noncommensurate if both prismatic and revolute joints occur (here ***w*** = **1** as we consider revolute joints only), ε is the step-width between the generated targets, and *t* indicates the time-step. ***q***^*home*^ is selected as a target with some probability *p*^*home*^≪1. The agent tries to reach and maintain each generated target ${\text{q}}_{t}^{*}$ using the online Goal Babbling basic scheme (GBSCHEME, cf. Algorithm 2) as following: The current inverse estimate for each generated target ${\text{q}}_{t}^{*}$ represents the motor torque ${\hat{\tau }}_{t}^{*}$ required to maintain this target. Correlated exploratory noise **σ** (Rolf et al., 2011) is added to discover and learn new configurations as specified in Equation (14) (cf. line 15 in Algorithm 2):

(14) $$\tau_{t}^{+}={\hat{\tau }}_{t}^{*}+\sigma (q_{t}^{*},t)$$

${\text{τ}}_{t}^{+}$ is the torque which is applied to the robot if ${\text{τ}}_{t}^{+}\in T_{s}$ holds or (if ${\text{τ}}_{t}^{+}\notin T_{s}$) it will be assigned to the nearest valid one (cf. line 16 in Algorithm 2), the outcome (${\text{q}}_{t}^{+}$) is then observed (cf. line 19 in Algorithm 2) and the inverse estimate is updated immediately (cf. line 21 in Algorithm 2). In simulation, a full dynamic simulation based on the forward dynamics model (Craig, 1986) of the robot is required.

The robot tries to explore along the desired direction until its actual direction of motion deviates from the intended one more than φ degrees. For $\varphi =\frac{\pi }{2}$, Equation (15) holds (cf. line 7 in Algorithm 2):

(15) $$\begin{array}{r} {\alpha =\left({\text{q}}_{t}^{*}-{\text{q}}_{t-1}^{*}\right)}^{T}\left({\text{q}}_{t}^{+}-{\text{q}}_{t-1}^{+}\right)<0 \end{array}$$

where ${\text{q}}_{t}^{+}$ is the currently observed configuration, ${\text{q}}_{t-1}^{+}$ is the previously observed one, ${\text{q}}_{t}^{*}$ is the generated target and ${\text{q}}_{t-1}^{*}$ is the previously generated one. In this case, the agent will return to its previous configuration ${\text{q}}_{t-1}^{+}$ to avoid drifting and start following a new randomly selected direction again (Rolf, 2013; Rayyes and Steil, 2016).

One criterion of the weighting scheme, which has been previously proposed in Rolf et al. (2011), is adopted in order to favor training samples:

(16) $$\begin{array}{r} w_{t}^{dir}=\frac{1}{2}\left(1+cos∢\left({\text{q}}_{t}^{*}-{\text{q}}_{t-1}^{*},{\text{q}}_{t}^{+}-{\text{q}}_{t-1}^{+}\right)\right) \end{array}$$

$w_{t}^{dir}$ is the direction criterion which evaluates whether the observed configuration and the generated target align well. This speeds up learning along the desired direction which is favorable in goal-directed algorithms. However, other weighting schemes could be selected as well.

### 5.3. Local linear map

As an incremental regression mechanism is required for online learning, a Local Linear Map (LLM) (Ritter, 1991) is employed. However, some modifications are necessary for exploiting symmetries. In this case, the learner must deal with scattered samples. Due to the initialization techniques of the standard LLM, receiving non-neighboring samples results in inconsistent outcomes. A further modification to gain more efficiency and reduce the number of required samples is proposed.

We will first explain the standard LLM algorithm for learning ISMs, and then the proposed modifications:

#### 5.3.1. LLM for learning ISMs

The inverse estimate ***Ĝ***(***q***) is initialized with a first local linear function ***Ĝ***^(1)^(***q***) which is centered around a prototype vector $q_{p}{}^{\left(1\right)}=q^{home}$ corresponding to the initial static torque ***τ***^*home*^. *M* different new local linear functions ***Ĝ***^(*i*)^(***q***) are added incrementally during learning, centered around prototype vectors $q_{p}{}^{\left(i\right)}$ and active only if new data is received in their close vicinity determined by a radius *d*. Let **ϱ**_*i*_ denote a local configuration vector given by Equation (17):

(17) $${\text{ϱ}}_{i}=\left(\frac{{\text{q}}^{*}-{\text{q}}_{\text{p}}{}^{\left(i\right)}}{d}\right)$$

The inverse estimate ***Ĝ***(***q***) is updated continuously and comprises a weighted linear sum of the linear functions ${\text{Ĝ}}^{\left(i\right)}\left({\text{ϱ}}_{i}\right)$. The weights are given by a Gaussian responsibility function *GR*(***q***) as shown in Equation (18).

(18) $$\begin{array}{l} \hat{\text{G}}({\text{q}}^{*})=\frac{1}{N(q^{*})}\sum_{i=1}^{M}GR(\varrho_{i})\cdot {\hat{G}}^{\left(i\right)}(\varrho_{i})\\ GR(\varrho )=\exp(-||\varrho |{|}^{2})\\ N(q^{*})=\sum_{i=1}^{M}GR(\varrho_{i})\\ {\hat{G}}^{\left(i\right)}(\varrho_{i})=W^{\left(i\right)}\cdot \varrho_{i}+o^{\left(i\right)},\; \end{array}\}$$

*N*(q^*^) normalizes the Gaussian responsibility functions in the inverse estimate.

The first linear function ***Ĝ***^(1)^(***q***) is initialized with $q_{p}{}^{\left(1\right)}=q^{home}$, **o**^(1)^ = ***τ***^*home*^, **W**^(1)^ = **0**, and ***Ĝ***^(1)^(***q***) = ***τ***^*home*^. A new local linear function ***Ĝ***^(*i*+1)^(***q***) will be added when the learner receives a new training sample ***q***_*new*_ at distance of at least *d* to all existing prototypes (i.e., $dist(q_{new},q_{p}{}^{\left(i\right)})⩾d$). The corresponding prototype vector is added ($q_{p}{}^{\left(i+1\right)}=q_{new}$). The offset **o**^(*i*+1)^ of ***Ĝ***^(*i*+1)^(***q***) is initialized with the inverse estimate before adding the new function in order to avoid abrupt changes in the inverse estimate function, i.e., the insertion of the new function will not change the local behavior of ***Ĝ***(***q***) at ***q***_*new*_. The weighting matrix **W**^(*i*+1)^ represents the slope of the linear function after inserting the new sample:

(19) $$\begin{array}{l} o^{\left(i+1\right)}=\hat{G}(q_{new}).\\ W^{\left(i+1\right)}=\frac{\partial \hat{G}(q)}{\partial q}=J(q) \end{array}\}$$

where *J*(***q***) is the Jacobian matrix of the inverse estimate (Rolf et al., 2011).

The parameter update is done at each step using a gradient descent with learning rate η in order to minimize the weighted squared error *E*_*t*_ given in Equation (21) as following:

(20) $$\begin{array}{l} W_{t+1}^{\left(i\right)}=W_{t}^{\left(i\right)}-\eta \;\cdot \;\frac{\partial E_{t}}{\partial {\text{W}}^{\left(i\right)}}\\ o_{t+1}^{\left(i\right)}=o_{t}^{\left(i\right)}-\eta \;\cdot \;\frac{\partial E_{t}}{\partial o^{\left(i\right)}} \end{array}\}$$

(21) $$E_{t}=w_{t}^{dir}\|\tau_{t}^{+}-{\hat{\tau }}_{t}^{+}{\|}^{2}$$

Note that the execution of ${\text{τ}}_{t}^{+}$ will result in ${\text{q}}_{t}^{+}$ and the corresponding torque estimated by the learner for ${\text{q}}_{t}^{+}$ is denoted by ${\hat{\tau }}_{t}^{+}$. Hence, the goal is to minimize the error between the executed and the estimated torques in order to improve the estimation accuracy.

The connections between the prototypes are organized and distributed based on an Instantaneous Topological Map (ITM) described in Jockusch and Ritter (1999) which is particularly suited to online map construction.

#### 5.3.2. LLM modifications

In this paper, two main modifications are implemented:

First, if the received new sample has a distance >2*d* to all existing prototypes, That causes a disproportionate change in the inverse estimate results due to the initialization techniques when inserting new functions (cf. Equation 19). The standard LLM therefore failed to approximate the model because of receiving non-neighboring samples when utilizing symmetries. To avoid such situations, the added function will be initialized with the new sample as given in Equation (22):

(22) $$\begin{array}{l} o^{\left(i+1\right)}=\tau_{new}\\ W^{\left(i+1\right)}=0 \end{array}\}$$

Second, the LLM approach updates the inverse estimate instantaneously and it therefore requires a lot of samples to converge. However, data acquisition is very costly in terms of time, tear, and wear. In order to reduce the number of required samples, multiple gradient descent steps are performed for each new sample until the error *E*_*t*_ stabilizes. Hence, each training sample has more influence on the inverse estimate update, and consequently, the number of required samples is reduced significantly.

### 5.4. The general scheme for symmetry discovery and learning ISMs

**Figure 8:**
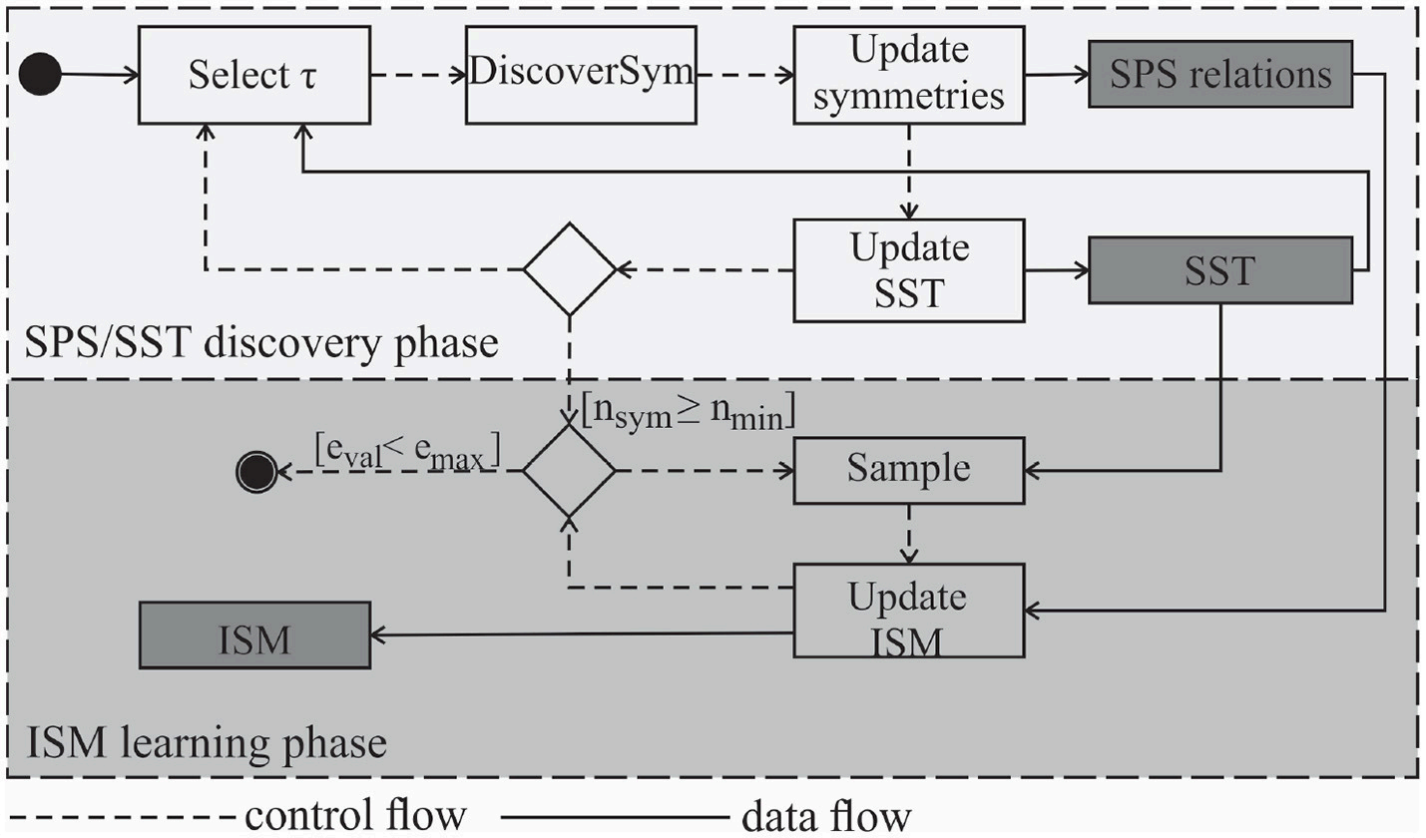
Flowchart of the **SST** and SPS discovery as well as the ISM learning phase. The estimated **SST** is used to generate admissible torque samples and the SPS is used to generate *n*_*sym*_ training samples from one recorded sample.

Figure 8 illustrates the required steps for symmetry discovery by generating torque profiles and for symmetry exploitation with online learning ISMs. In the discovery phase, first a target torque ***τ*** is selected. Subsequently, Algorithm 1 is applied to discover symmetric configurations. Multiple linear regression is then performed using the output of Algorithm 1 to update the functional relations between primary symmetries. The applied torque profiles and observed joint angles are exploited to update the estimates of the **SST** and optionally the **BCTS** (cf. section 4.3). When a sufficient number of primary symmetries *n*_*sym*_≥*n*_*min*_ of symmetries has been discovered, the learning phase begins and the functional relations between the primary symmetries are exploited to generate *n*_*sym*_ training samples based on one applied training torque vector. *n*_*min*_ is set here to the number of geometrical symmetries. Constrained Direction Sampling (cf. Algorithm 2) or any other online (or batch) learning approach can be applied to obtain the ISM. The learning phase is terminated if a desired validation error *e*_*max*_ (i.e., the torque RMSE threshold) is reached. *e*_*max*_ is determined based on the torque limits and the required accuracy for accomplishing the task. *e*_*val*_ is the training torque RMSE which is evaluated at each iteration (i.e., predefined number of samples) on randomly chosen training samples from the current iteration.

**Figure 9:**
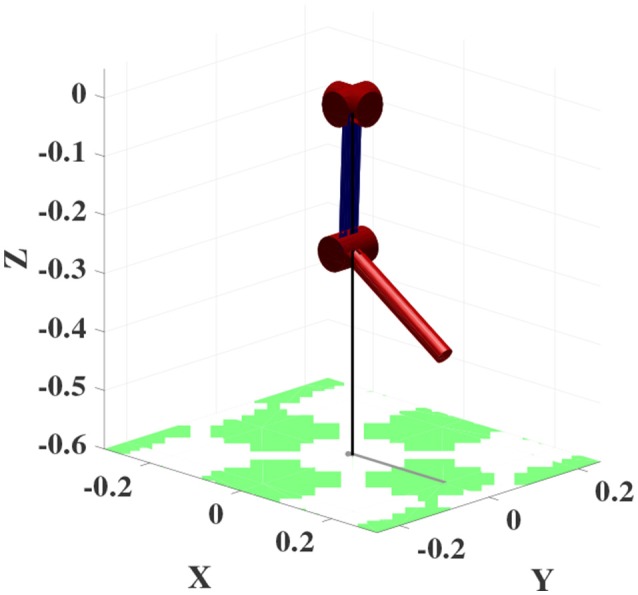
Structure of the 3R simplified human arm with 25*cm* link length.

## 6. Batch learning

Lattice sampling is implemented to sample the **BCTS** and collect training data. A feed-forward network with *n* neurons in the hidden layer is implemented to learn ISMs in a batch learning fashion.

A lattice $L_{s}$ is the set of points which is characterized by an elementary unit cell. This elementary unit cell can be described by *m* vectors given in Equation (23) and is replicated over *m*-dimensional space.

(23) $${ℒ}_{s}=\sum_{i=1}^{m}\lambda_{i}\cdot \;p_{i},\;0⩽\lambda_{i}⩽1$$

The vectors **p**_*i*_ are called also generators of the lattice (Cervellera et al., 2014).

## 7. Experimental results

This section presents experimental results for learning ISMs for a 2R planar robot and a 3R simplified human arm (Babiarz et al., 2015). The results show the efficiency gained by exploiting symmetries and demonstrate that exploiting symmetries is a generally applicable strategy which can be utilized with offline/online learning algorithms. Moreover, we demonstrate the efficiency gained by implementing LLM with multiple gradient descent steps (cf. section 5.3.2) for a 2R planar robot.

### 7.1. Exploiting symmetries with constrained direction sampling - online learning

#### 7.1.1. 2R planar manipulator

**Figure 10:**
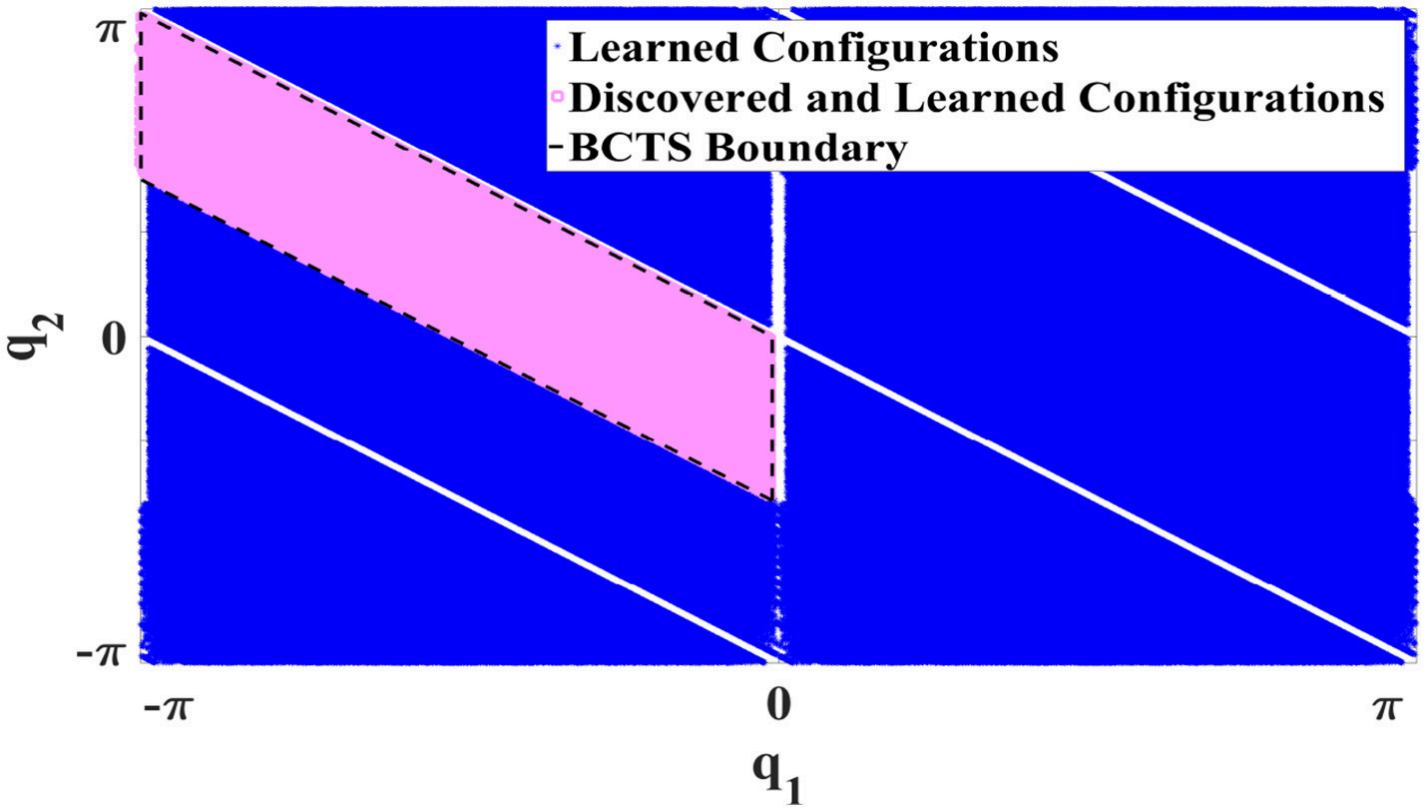
Explored configurations (red) and learned configurations (blue) for the 2R robot by exploiting symmetries using Constrained Direction Sampling and LLM.

Constrained Direction Sampling was employed to explore the **BCTS** and learn the ISM for the entire configuration space of the 2R planar robot (cf. Figure 2A) for which, each link length is 25*cm*. Figure 10 shows the learned area of the configuration space (blue area) by exploring merely the **BCTS** (red area) and exploiting the symmetries.

**Figure 11:**
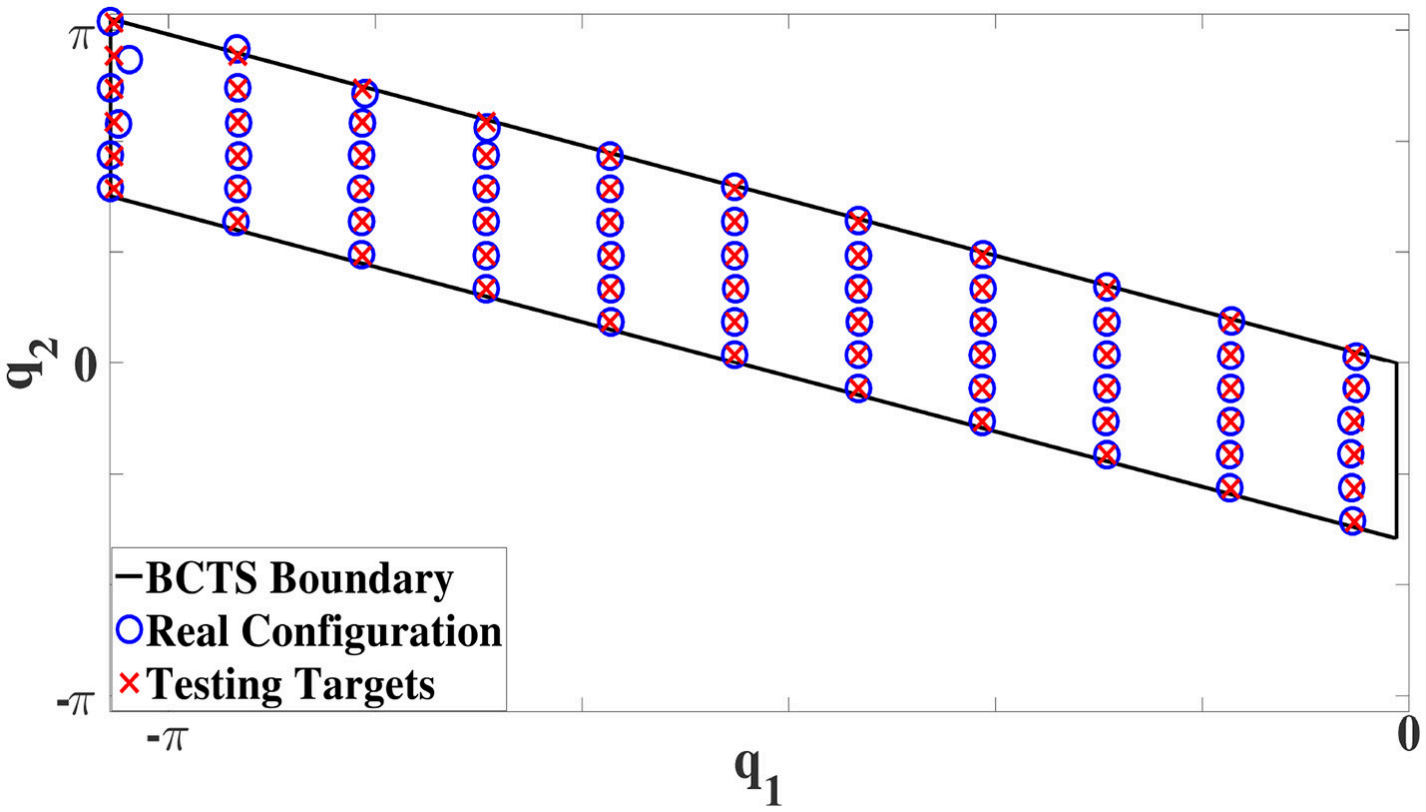
Test performance for the 2R robot. The ISM is learned utilizing Constrained Direction Sampling and LLM with an RMSE of 0.0053*Nm*. The boundary of the **BCTS** is indicated by the black parallelogram, the red crosses indicate the test targets, and the blue circles represent the observed configurations.

**Figure 12:**
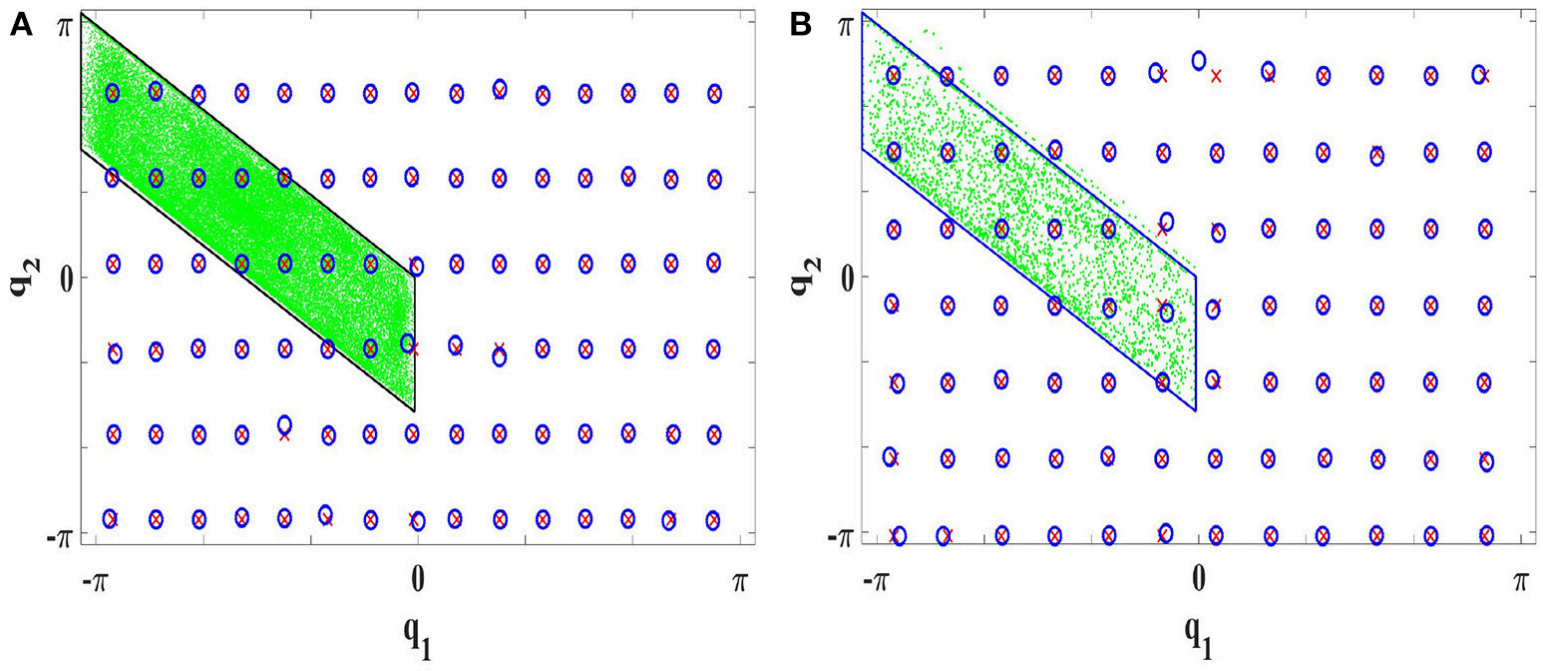
Constrained Direction Sampling results for the 2R planar robot utilizing **(A)** LLM with 540 iterations **(B)** LLM_*it*_ with 30 iterations. Torque RMSE is 0.0052*Nm*. The green area is the discovered **BCTS**, the red crosses are the test targets, and the blue circles represent the real observed configurations.

After the training phase, the robot tries to reach and maintain 66 configuration targets regularly distributed on a grid in the **BCTS**. All targets were maintained well with an RMSE of 0.0053*Nm* which represents the difference between the learner output, i.e., the estimated torque and the actual required static torque. Compared to the minimum and maximum static torques (−18.4, 24.5)*Nm* and (−6, 6.2)*Nm* for the first and second joints, respectively, the observed RMSE is negligibly small. Figure 11 illustrates the results in the configuration space. The red crosses indicate the targets, and the blue circles represent the observed configurations which illustrate the good performance as well; the boundary of the **BCTS** is indicated by the black parallelogram. Subsequently, the robot tries to maintain another 90 targets scattered over the entire configuration space. The performance was also very good, the robot managed to achieve all targets very accurately with an RMSE of 0.0052*Nm* as shown in Figure 12.

##### Efficiency gained by iterating gradient descent step in LLM:

**Figure 13:**
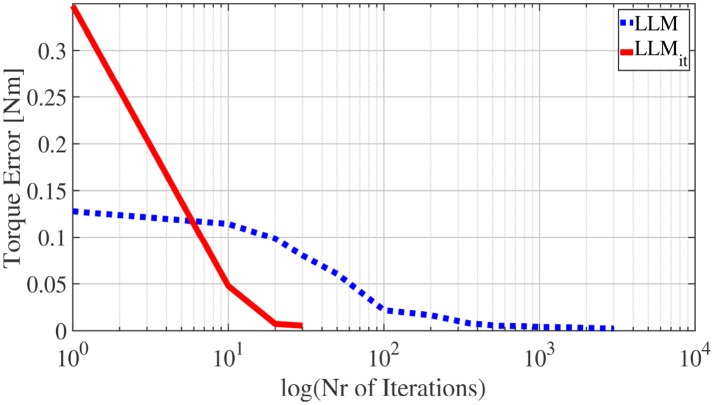
Torque RMSE for Constrained Direction Sampling with LLM_*it*_ (red) and LLM (blue).

In the experiment, LLM with a single gradient descent step per sample was implemented first with Constrained Direction Sampling. At least 540 iterations (each iteration consists of 100 samples) were required to discover the entire **BCTS** and achieve an RMSE of 0.0053*Nm*. By increasing the number of iterations, the performance accuracy is increased as shown in Figure 13. The blue line represents the RSME of the torque evaluated for different numbers of iterations. The RMSE was 0.0024*Nm* after 3000 iterations.

A significant reduction in the number of required samples was observed by iterating multiple gradient descent steps in LLM (LLM_*it*_) with Constrained Direction Sampling. Only 30 iterations were required to learn the ISM and achieve the same accuracy, i.e., test RMSE of 0.0053*Nm*. Hence, the number of required samples are decreased by a factor of 18. The robot performance is tested on 84 targets scattered over the entire configuration space as shown in Figure 12B.

The average training time required in each iteration for updating the LLM_*it*_ is 3*min* and 0.2*min* for the LLM. Hence, the time cost per iteration for LLM_*it*_ is 15 times higher. However, LLM requires 18 times the number of samples required for LLM_*it*_. As data acquisition is costly and moving the robot to the sampled configurations is very time-consuming, the overall efficiency with LLM_*it*_ is much higher than with LLM.

The torque RMSEs for different numbers of iterations (red line) are shown in Figure 13. As we can see from the figure, the torque RMSE converges much faster for LLM_*it*_ than LLM.

#### 7.1.2. 3R robot arm

Constrained Direction Sampling with LLM_*it*_ is implemented to learn the ISM for the 3R manipulator (cf. Figure 9). After exploring the **BCTS**, the robot performance is tested on 64 targets regularly distributed on a grid in the configuration space. At least 140 iterations were required to achieve an RMSE of 0.26*Nm*. The minimum and maximum torques for the first, the second, and the third joints are (−24.4, 24)*Nm*, (−24.2, 24.2)*Nm*, and (−12.4, 12.2)*Nm*, respectively. The achieved accuracy is very good compared to the torque limits. The results are illustrated in the configuration space as shown in Figure 15A.

### 7.2. Exploiting symmetries with lattice sampling - batch learning

#### 7.2.1. 2R planar manipulator

**Figure 14:**
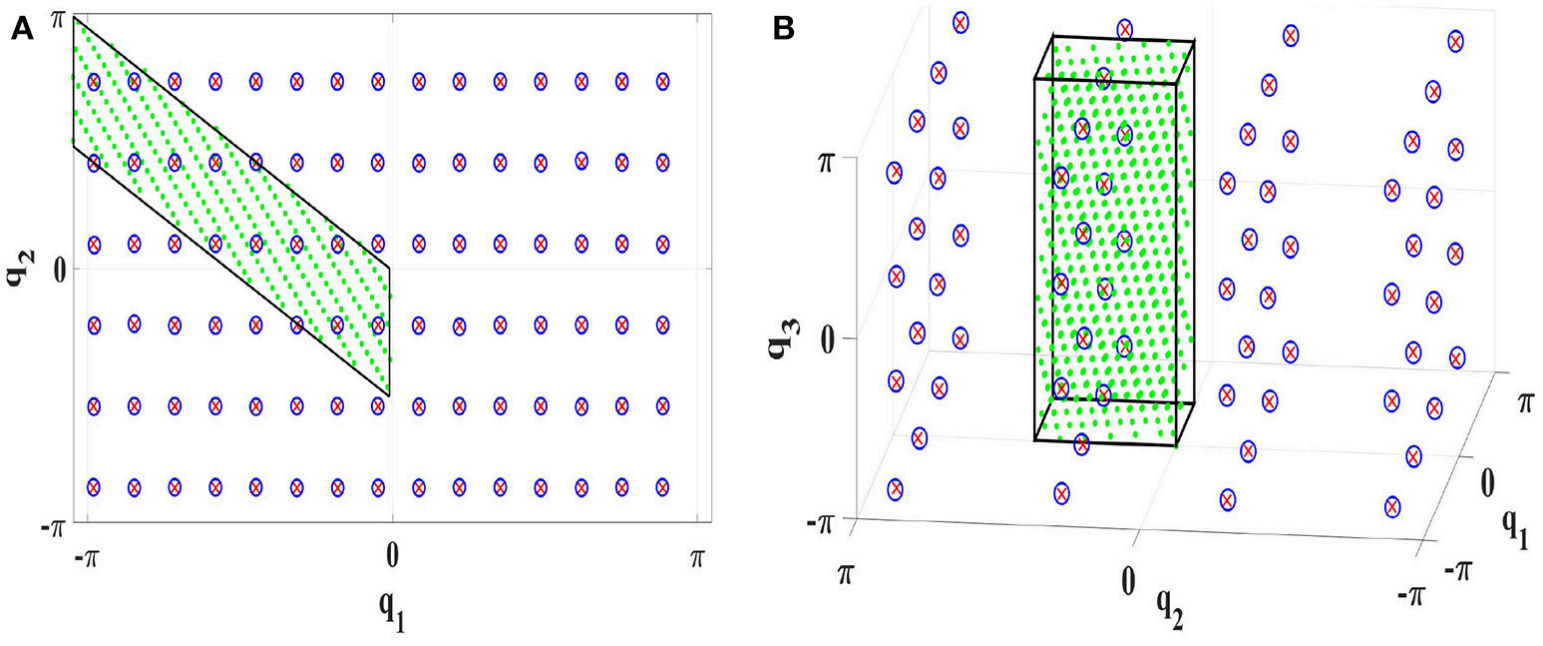
Learning ISMs with exploiting symmetries with batch learning **(A)** for the 2R planar manipulator with an RMSE of 0.0051*Nm*** (B)** for the 3R manipulator with an RMSE of 0.009*Nm*. The green area represents the discovered **BCTS**, the border of the **BCTS** is indicated by the black lines, the test targets are visualized by red crosses and the blue circles indicate the real configurations.

To demonstrate the general applicability of symmetry exploitation, we investigate batch learning to learn the ISM of the 2R robot (cf. Figure 2A) based on a lattice sampling approach. Lattice sampling was performed to collect training samples in the **BCTS**. A feed-forward neural network with one hidden layer consisting of 18 neurons was used in a batch learning fashion. Only 255 samples in the **BCTS** were required to learn the ISM for the entire configuration space with almost the same testing torque RMSE of 0.0051 using the same 90 testing targets as in section 7.1.1. The result is illustrated in Figure 14A.

Lattice sampling was then performed for the entire configuration space without exploiting symmetries. 2040 samples were required to achieve approximately the same RMSE of 0.005*Nm*. The number of required samples to learn the ISM of the 2R robot was reduced by a factor of 8 by exploiting primary symmetries. This factor corresponds well to the number of 8 primary symmetries for the 2R robot.

#### 7.2.2. 3R robot manipulator

**Figure 15:**
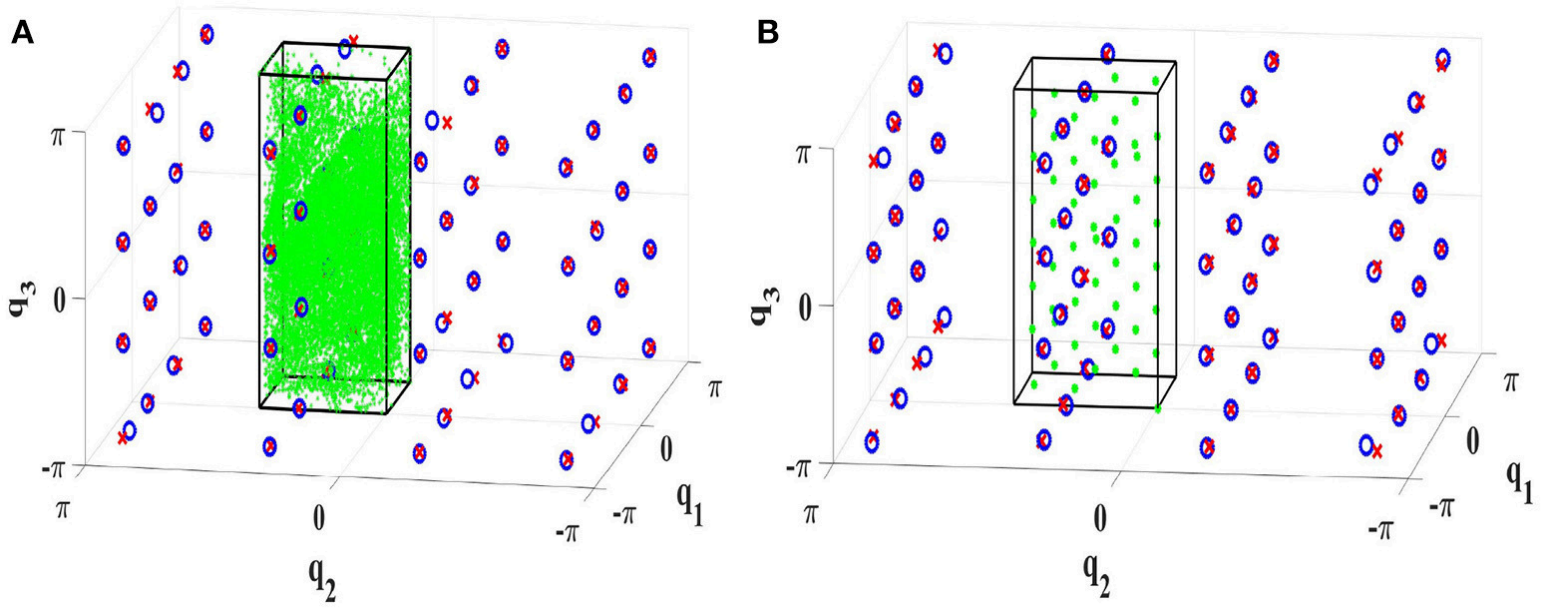
Learning ISMs with exploiting symmetries **(A)** online with Constrained Direction Sampling **(B)** offline with Lattice Sampling. The torque RMSE is 0.028*Nm*. The green area represents the discovered **BCTS**, the border of the **BCTS** is indicated by the black cube, the test targets are visualized by red crosses and the blue circles indicate the real configurations.

We did the same experiment as in section 7.1.2 utilizing lattice sampling and a feed-forward neural network with 18 neurons in the hidden layer in offline learning fashion. Only 65 training samples in the **BCTS** were required to achieve approximately the same accuracy with RMSE of 0.28*Nm*. The good performance of the robot is also illustrated in Figure 15B. To illustrate the efficiency gained by using symmetries, Lattice sampling was implemented without exploiting symmetries. 855 samples were required to explore the entire configuration space with approximately the same RMSE of 0.03*Nm*. The number of required samples to learn the ISM of the 3R robot was reduced by a factor of 16.13 which matches the number of 16 primary symmetries well. To achieve higher accuracy, 600 samples with 30 hidden neurons were required to achieve an RMSE of 0.009*Nm*. The result is demonstrated in Figure 14B.

### 7.3. Discussion

The number of required samples to learn ISMs for 2R and 3R manipulators were reduced by a factor of 8 and 16, respectively, resulting from exploiting primary symmetries and constraining the exploration to the **BCTS** only. Hence, exploiting symmetries can drastically increase learning efficiency – regardless whether offline or online learning schemes are considered – by reducing the number of required samples by a factor which approximately equals the number of discovered primary symmetries in the presented experiments. Further efficiency gains can be expected if secondary symmetries are exploited as well.

Note that the number of samples in batch learning is lower than that required in the presented online learning approach. Nevertheless, even batch learning approaches can greatly benefit from a significant reduction in the number of required samples by exploiting symmetries. However, online learning techniques such as Goal Babbling and Direction Sampling, which generate targets on the fly and update the learner at each step simultaneously, best fit the concepts of gradual exploration as well as “learning while behaving” – hence they best reflect human developmental aspects in robot learning.

## 8. Conclusion and outlook

We showed that inverse statics mappings of discretely-actuated serial manipulators can be learned very accurately, if the problems arising from exploratory learning in the torque domain are properly addressed. To learn ISMs online and from scratch, we constrained the Direction Sampling approach and improved the LLM learner. Naturally, these modifications may be useful also in other contexts and comprise a contribution to increase efficiency of any learning scheme employing these methods. Moreover, we demonstrated that the efficiency of learning inverse statics mappings can be further increased significantly by exploiting inherent symmetries of the mapping, a concept that we formalized properly and which as well is relevant beyond the particular exploratory learning application. To demonstrate its generality, we successfully integrated it into online Constrained Direction Sampling and a more standard batch learning approach based on lattice sampling. The presented results indicated that factors of at least 8 and 16 w.r.t. the number of samples can be achieved for a 2R and a 3R robot, respectively. Thus, exploiting symmetries is a promising strategy to increase the efficiency of learning both online and offline, and it is rather a general strategy and not limited to learning ISMs only, but it can be exploited in other functions or mappings.

We initially considered the particular problem of learning the inverse statics model as a rather simpler subproblem of the general inverse dynamics exploratory learning. However, it appears that it already displays some major difficulties of torque-based exploratory learning. And it requires substantial effort to be tackled. That led to the novel approaches on symmetries and the learning methods presented in this paper, which all have their right in itself and provide useful tools beyond the ISM learning alone. It is not obvious though, how to make the next step toward general inverse dynamics exploratory learning without relying on a pre-defined closed-loop controller, because that requires to suggest a general way to automatically choose target trajectories in the joint space that are safe, but representative and increasingly complex, while all other problems of efficiency and ambiguity still remain.

Currently, our approach is limited to primary symmetries as the functional relations between secondary symmetries prove to be challenging. Furthermore, elasticity as well as nonlinear friction effects are currently not considered. This sheds some light on more direct and natural extensions for future work, which we are working on. The proposed symmetry-based exploration is being (i) implemented in the real application, (ii) generalized to learn primary and secondary symmetries for discretely-actuated serial manipulators with arbitrary geometrical and inertial properties, (iii) extended to incorporate link and joint flexibility as well as nonlinear friction effects, which will pave the way for thorough experimental evaluation on a robot with variable stiffness actuators and (iv) implement a dictionary with a fixed budget to update LLM using a sub-data set instead of the current sample only. Furthermore, due to the same dimensionality of action and observation spaces, the efficiency advantage of Goal Babbling is less pronounced for learning ISMs than learning IK. However, this disadvantage is partially compensated by the efficiency gained by exploiting symmetry properties of ISMs and limiting the exploration to **BCTS** only. In our recent work (Rayyes et al., 2018), we additionally lay the foundation for increasing the scalability by learning IK and the inverse statics IS_*x*_ and ISMs simultaneously. IS_*x*_ maps from Cartesian space to the motor space. Hence, ISMs can be inferred by relating IK and IS_*X*_.

## Author contributions

RR: Conception and design of the research, acquisition, analysis and interpretation of data; DK: Conception and design of the research, analysis and interpretation of data; JS: Conception and design of the research, analysis and interpretation of data. All authors writing of the paper.

### Conflict of interest statement

The authors declare that the research was conducted in the absence of any commercial or financial relationships that could be construed as a potential conflict of interest.

## Footnotes

^1^Obviously, in practical applications, a software joint limit is employed to avoid reaching the hardware joint limit.

## References

[B1] Akiyama T. Hachiya H. Sugiyama M. (2010). Efficient exploration through active learning for value function approximation in reinforcement learning. Neural Netw. 23, 639–648. 10.1016/j.neunet.2009.12.010 20080026

[B2] Asada M. MacDorman K. F. Ishiguro H. Kuniyoshi Y. (2001). Cognitive developmental robotics as a new paradigm for the design of humanoid robots. Rob. Auton. Syst. 37, 185–193. 10.1016/S0921-8890(01)00157-9

[B3] Babiarz A. Czornik A. Klamka J. Niezabitowski M. (2015). Dynamics modeling of 3d human arm using switched linear systems. Asian Conference on Intelligent Information and Database Systems (Cham: Springer), vol. 9012, 258–267.

[B4] Baranes A. Oudeyer P. (2013). Active learning of inverse models with intrinsically motivated goal exploration in robots. Robot. Auton. Syst. 61, 49–73. 10.1016/j.robot.2012.05.008

[B5] Cervellera C. Gaggero M. Macciò D. Marcialis R. (2014). Lattice sampling for efficient learning with nadaraya-watson local models in 2014 International Joint Conference on Neural Networks (IJCNN) (Beijing: IEEE), 1915–1922.

[B6] Craig J. (1986). Introduction to Robotics: Mechanics & Control. Boston, MA: Addison-Wesley Publishing.

[B7] De Luca A. Panzieri S. (1994). An iterative scheme for learning gravity compensation in flexible robot arms. Automatica 30, 993–1002.

[B8] De Luca A. Panzieri S. (1996). End-effector regulation of robots with elastic elements by an iterative scheme. Int. J. Adapt. Control Signal Proc. 10, 379–393.

[B9] Demiris Y. Meltzoff A. (2008). The robot in the crib: a developmental analysis of imitation skills in infants and robots. Infant Child Dev. 17, 43–53. 10.1002/icd.543 18458795 PMC2367332

[B10] Draper N. R. Smith H. (1998). Applied Regression Analysis. New York, NY: Wiley.

[B11] D'Souza, Vijayakumar S. Schaal S. (2001). Learning inverse kinematics. Int. Conf. Intell. Rob. Syst. 1, 298–303. 10.1109/IROS.2001.973374

[B12] Forestier S. Oudeyer P. (2016). Modular active curiosity-driven discovery of tool use in 2016 IEEE/RSJ International Conference on Intelligent Robots and Systems, IROS 2016 (Daejeon), 3965–3972.

[B13] Giorelli M. Renda F. Calisti M. Arienti A. Ferri G. Laschi C. (2015). Neural network and jacobian method for solving the inverse statics of a cable-driven soft arm with nonconstant curvature. IEEE Trans. Robot. 31, 823–834. 10.1109/TRO.2015.2428511

[B14] Gomi H. Kawato M. (1993). Neural network control for a closed-loop system using feedback-error-learning. Neural Netw. 6, 933–946. 10.1016/S0893-6080(09)80004-X

[B15] Jockusch J. Ritter H. (1999). An instantaneous topological mapping model for correlated stimuli in Neural Networks, 1999. IJCNN '99. International Joint Conference (Washington, DC), vol. 1, 529–534.

[B16] Jordan M. Rumelhart D. (1992). Forward models: supervised learning with a distal teacher. Cogn. Sci. 16, 307–354.

[B17] Loviken P. Hemion N. (2017). Online-learning and planning in high dimensions with finite element goal babbling. Joint IEEE International Conference on Development and Learning and Epigenetic Robotics (ICDL-EpiRob) (Lisbon).

[B18] Luca A. D. Panzieri S. (1993). Learning gravity compensation in robots: rigid arms, elastic joints, flexible links. Int. J. Adapt. Control Signal Proc. 7, 417–433.

[B19] Meier F. Kappler D. Ratliff N. Schaal S. (2016). Towards robust online inverse dynamics learning in 2016 IEEE/RSJ International Conference on Intelligent Robots and Systems (IROS), (Daejeon), 4034–4039.

[B20] Moulin-Frier C. Nguyen S. M. Oudeyer P.-Y. (2013). Self-organization of early vocal development in infants and machines: The role of intrinsic motivation. Front. Psychol. 4:1006. 10.3389/fpsyg.2013.01006 24474941 PMC3893575

[B21] Peters J. Schaal S. (2008). Learning to control in operational space. Int. J. Robot. Res. 27, 197–212. 10.1177/0278364907087548

[B22] Philippsen A. K. Reinhart R. F. Wrede B. (2016). Goal babbling of acoustic-articulatory models with adaptive exploration noise in 2016 Joint IEEE International Conference on Development and Learning and Epigenetic Robotics (ICDL-EpiRob) (Cergy-Pontoise: IEEE), 72–78.

[B23] Rayyes R. Kubus D. Steil J. (2018). Multi-stage goal babbling for learning inverse models simultaneously in IROS Workshop 2018, BODIS: The Utility of Body, Interaction and Self Learning in Robotics Workshop (Madrid).

[B24] Rayyes R. Steil J. J. (2016). Goal babbling with direction sampling for simultaneous exploration and learning of inverse kinematics of a humanoid robot in Proceedings of the Workshop on New Challenges in Neural Computation, Vol. 4 (Hanover), 56–63.

[B25] Ritter H. (1991). Learning with the self-organizing map in Artificial Neural Networks : Proceedings of the 1991 International Conference on Artificial Neural Networks [ICANN-91], vol. 1, ed Kohonen T. (Espoo), 379–384.

[B26] Rolf M. (2013). Goal babbling with unknown ranges: a direction-sampling approach in IEEE International Conference on Development and Learning and Epigenetic Robotics (ICDL) (Osaka), 1–7.

[B27] Rolf M. Steil J. (2014). Efficient exploratory learning of inverse kinematics on a bionic elephant trunk. IEEE Trans. Neural Netw. Learn. Syst. 25, 1147–1160. 10.1109/TNNLS.2013.2287890

[B28] Rolf M. Steil J. J. Gienger M. (2010). Goal babbling permits direct learning of inverse kinematics. IEEE Trans. Auton. Mental Dev. 2, 216–229. 10.1109/TAMD.2010.2062511

[B29] Rolf M. Steil J. J. Gienger M. (2011). Online goal babbling for rapid bootstrapping of inverse models in high dimensions in IEEE International Conference on Development and Learning (ICDL) (Frankfurt am Main), 1–8.

[B30] Şimşek O. Barto A. G. (2006). An intrinsic reward mechanism for efficient exploration in Proceedings of the 23rd International Conference on Machine Learning, ICML '06, (New York, NY: ACM), 833–840.

[B31] Thuruthel T. G. Falotico E. Cianchetti M. Laschi C. (2016a). Learning global inverse kinematics solution for a continuum robot in Robot Design and Control. ROMANSY21, Vol. 569, eds Parenti-Castelli V. Schiehlen W. (Cham: Springer), 47–54.

[B32] Thuruthel T. G. Falotico E. Cianchetti M. Renda F. Laschi C. (2016b). Learning global inverse statics solution for a redundant soft robot in Proceedings of the 13th International Conference on Informatics in Control, Automation and Robotics (Lisbon: SciTePress), 303–310.

[B33] Vijayakumar S. D'Souza A. Schaal S. (2005). Incremental online learning in high dimensions. Neural Comput. 17, 2602–2634. 10.1162/089976605774320557 16212764

[B34] von Hofsten C. (1982). Eye Hand Coordination in the Newborn. Washington, DC: American Psychological Association, 450–461.

[B35] Wolpert D. Kawato M. (1998). Multiple paired forward and inverse models for motor control. Neural Netw. 11, 1317–1329. 12662752 10.1016/s0893-6080(98)00066-5

[B36] Wolpert D. Miall R. C. Kawato M. (1998). Internal models in the cerebellum. Trends Cognit. Sci. 2, 338–347. 21227230 10.1016/s1364-6613(98)01221-2

[B37] Xie M. Zhong Z. W. Zhang L. Yang H. J. Song C. S. Li J. (2008). Self learning of gravity compensation by loch humanoid robot. International Conference on Humanoid Robots (Daejeon), 320–325.

